# Differentiation of specific wear rates of AISI 304 austenitic and AISI 2205 duplex stainless steels at room and high temperatures

**DOI:** 10.1016/j.heliyon.2022.e11807

**Published:** 2022-11-23

**Authors:** Dler A. Ahmed, Mohammedtaher M. Mulapeer

**Affiliations:** a Department of Mechanical &Mechatronics, College of Engineering, Salahaddin University - Erbil, Erbil Polytechnic University, Iraq; b Department of Mechanical &Mechatronics, College of Engineering, Salahaddin University - Erbil, Iraq

**Keywords:** Austenite stainless steel, Duplex stainless steel, Specific wear rate, High temperature, Contact temperature, Oxide phase

## Abstract

Austenitic and duplex stainless steels are used in numerous applications at room and high temperatures. However, their wear behaviour at high temperatures has not yet been sufficiently investigated. Therefore, in this work, wear tests were carried out on specimens of AISI 304 austenitic steels and AISI 2205 duplex stainless steels at temperatures (30 °C, 140°, 300 °C, 460 °C, and 570 °C). It was found that the specific wear rate of AISI 304 at room temperature was only 6% higher than that of AISI 2205, but this ratio increased and exceeded 50% at 460 °C and 570 °C. According to scanning electron microscopy and X-ray diffraction, oxide formation on the worn surfaces started at 300 °C and increased with increasing temperature. The oxide layers protected the surface and reduced the wear and material removal. The contact temperature between the sliding surfaces was significantly higher than the ambient temperature and was responsible for the oxide formation on the surfaces. The results indicated that the AISI 2205 exhibited better wear resistance than the AISI 304 at high temperatures.

## 1 Introduction

Among stainless steels (ASS), austenitic grades, and specially AISI 304 has the most used in bearings, chemical industries, and surgical and marine equipment (Prajapati and Tiwari, 2021; khaleal et al., 2020). Although their excellent corrosion resistance, their low yield strength restricts applications in harsh environments and may necessitate searching for a suitable substitute (Avilaa et al., 2020; Alabdullah et al., 2016; Moharana et al., 2016). However, it is used in conditions that exposure to high temperatures such as heat exchangers and refinery accessories, (Comittee of Stainless Steel Producers, 2015). Duplex stainless steel (DSS) grades due to their dual phases of ferrite-austenite have moderate corrosion resistance and high strength and hardness (Lindgren et al., 2015). Therefore, they are used in important applications such as petrochemical industries, mining processes, and nuclear plans (Zhu et al., 2018; Tahchieva et al., 2019). AISI 2205 is also recommended by researchers as a replacement for austenitic alloys due to its higher mechanical properties and lower nickel content (Kocijan et al., 2011; Jebaraj and Ajaykumar, 2013). As a result, it is critical to compare the wear behavior of both alloys under different conditions to determine which is superior in a given application.

Wear behavior is a material phenomenon and specific wear rate (SWR) is one of the most important surface properties for selecting materials that are susceptible to friction and wear (Rababa and Al-Mahasne, 2011; Suthar et al., 2015). Because wear reduces the service life of components and directly affects the economy. For example, wear is a major problem in mineral processing industries, which occurs in tools and machine parts (Li et al., 2019; Monga et al., 2018). A great deal of work has been done on the mechanical properties and surface quality of ASS and DSS (Karahan et al., 2014; Oke et al., 2018). Wear behavior has also recently attracted the interest of researchers, however, most of them have studied wear at room temperature, while in various applications such as engines, refineries, and the food industry, wear occurs at high temperatures (Alvi et al., 2020; Ge et al., 2019).

Scholars such as Chawla et al. (2013) and Rokanopoulou et al. (2014) investigated the effect of applied load and sliding speed on the wear rate of AISI 304 and AISI 2205 at room temperature. Others such as Singh and his colleagues (Singh et al., 2019) improved wear resistance of AISI 304 by heat treatment and formation of oxide layers then wear was tested at room temperature.

There are some works were done on the wear rate at high temperatures for specific materials. For example, Parthasarathi et al. (2013) observed that the coefficient of friction and surface roughness of austenitic stainless steel increased with the increase in temperature. Bayata and Alpas (2020) investigated the wear behavior of NCF-3015 and Inconel-751 superalloys at high temperatures. They found that the wear rate increased with the increase of temperature up to 350 °C and then decreased significantly, due to the formation of different types of oxides. Parthasarathi and Duraiselvam (2010) studied the wear rate of austenitic stainless steel at high temperatures and claimed that the oxide layer began to form at 350 °C and more. Kennedy et al. (2015) found that the contact temperature is higher than the ambient temperature according to the test condition, and this high temperature causes the reaction and oxide formation.

Based on previous literature, the wear investigation of ASS and DSS at room temperature has been adequately conducted. However, very little is currently known about the wear rate of AISI 304 and AISI 2205 at high temperatures, and the comparison between them. The novelty of this work is the study of SWR at room and high temperatures of AISI 304 and AISI 2205. This research aims to emulate the actual wear rate that occurs in engines and refineries at different temperatures and determine which of the two is more accurate.

The temperature range 30 °C–570 °C was chosen based on the range of previous literature, e.g.: Alvi et al. (2020) worked from RT to 600 °C, Hernandez et al. (2014) from 20 °C to 400 °C, Parthasarathi et al. (2013) from RT to 550 °C, and Bayata and Alpas (2020) from 25 °C to 650 °C. Scanning electron microscopy (SEM) was used to illustrate the morphology of the worn surfaces, and Xpert high score software was used to measure and interpret the unique diffractions obtained from the X-ray diffraction (XRD) tests.

## 2 Experimental procedure

The chemical composition of the pins and discs was determined using a metal analyzer model from Spectromaxx Spectro Company. In addition, the ultimate strength of the alloys was tested using the Universal Testing Machine model TERCO MT 3037, with a specimen diameter of 6 mm and a gauge length of 24 mm according to the ASTM E8 (2010). Furthermore, Vickers hardness was measured using AKASHI testing machine model AVK with a load of 30 kg. f and a time of 15 s under ASTM E92-17 (1997). Samples were etched according to the ASTM E7 (2012) then the optical microscope Carl Zeiss Axiovert 25 was used to investigate the microstructure of the materials used. In addition, SEM images and XRD technique were done to inspect the morphology and composite of the worn surfaces.

In this work, a sample grinding machine was used as a wear test machine after adding a cylindrical furnace, temperature-controlled, and a load cell to measure normal load. A 1000 W circular heater was installed inside the furnace, as well as a vertical rotating shaft to carry the disc. To measure the inside temperature of the furnace and near the pin, a K-type thermocouple was installed in the center of the cover. Following ASTM G 99 (2012), Sampath (2015), S. T. Method (2010), pins of Ø10 × 70 mm length and discs of Ø120 × 4 mm thickness with an average contact diameter of 85 mm were prepared. The pins were polished and the surface of the discs had an average accurate surface finish of 0.7 μm. After that, specimens were cleaned with acetone to remove any particles between the sliding surfaces and to ensure metal contact during the wear tests.

To focus on the effect of temperature, in this work, the operation parameters of applied load, sliding speed, and siding distance were set to 45 N, 1.34 m/s, and 2000 m, respectively since these factors had already been studied extensively. Five temperature levels were used, from room temperature to 570 °C. The amount of material removal was calculated from the difference in mass of the pins before and after the tests, using Precisa XB 220 A balance with an accuracy of 0.0001 g. Volumetric wear was calculated by dividing the difference of mass by the density of the stainless steel (Parthasarathi and Duraiselvam, 2010).

To measure temperatures at the sliding surfaces, a thermocouple sensor was inserted into the unhallowed drilled pin 1.0–2 mm from the end of the pin. The experiments were repeated to determine the surface temperature. Each test lasted 13 min to ensure that the maximum contact temperature was obtained. The temperature sharply climbed within the first minute, then the change was very slightly increased after 2 min and the surface contact temperature remained constant at the highest value after 5 min as can be seen from the data in Table 1.

**Table 1:** Experimental matrix with volume wear results of AISI 304 and AISI 2205.

No.	Temp (°C)	Load (N)	Speed (rpm)	AISI 304 Wear (mm^3^)	AISI 2205 Wear (mm^3^)	AISI 304 T_max_ (°C)	AISI 2205 T_max_ (°C)	Average. T_max_ (°C)
1.	30	45	1.34	28.4056	26.67092	228	208	218
2.	140	45	1.34	45.749	40.3061	275	287	281
3.	300	45	1.34	37.2194	23.9211	445	455	450
4.	460	45	1.34	17.786	2.8698	561	556	564
5.	570	45	1.34	2.5765	1.1862	681	661	671

SWR is more accurate than volumetric wear when studying the wear behavior of the materials according to previous works such as Davanageri et al. (2018), Abreu et al. (2015) and Davanageri et al. (2019). Therefore, SWR is calculated by dividing the wear volume by the applied load and sliding distance according to the following equation (Ahmed and Mulapeer, 2021; Marques et al., 2011).

(1) SWR=W/(D.L)

where W is the volume of removal material in mm^3^, D is the sliding distance which is constant at 2000 m in this work, and L is the applied load in N.

## 3 Results

**Figure 1:**
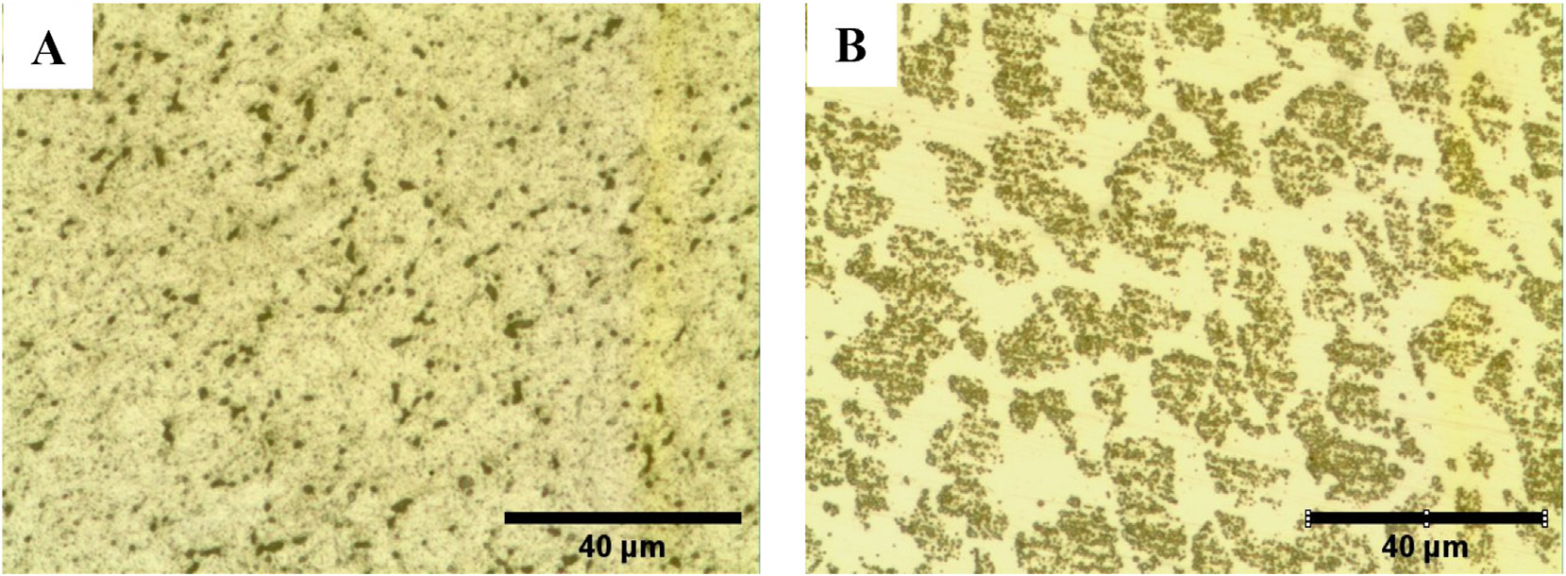
Microstructure of as received A) AISI 304 austenitic and B) AISI 2205 duplex stainless steel with Magnification of X 500.

Table 2 shows that as the chromium and molybdenum content increases and the nickel content decreases, the hardness and ultimate strength of the alloys increase significantly. The microstructure of AISI 304 had austenite grains with a small amount of ferrite distributed in grain boundaries, while the microstructure of AISI 2205 was almost equally composed of ferrite and austenite, as shown in Figure 1.

**Table 2:** Chemical composition of stainless steel AISI 304, AISI 2205, and AISI 2507.

AISI	C	Cr	Ni	Mo	Cu	Co	Ultimate Strength (Mpa)	Hardness (HV)
304	0.074	17.23	9.79	0.313	0.481	0.293	766.2	303.4
2205	0.109	22.42	4.75	2.88	0.075	0.135	997.9	321.4
2507	0.065	23.46	5.16	2.93	0.065	0.133	1146	407.3

### 3.1 Effect of temperature on the specific wear rate and contact temperature

**Figure 2:**
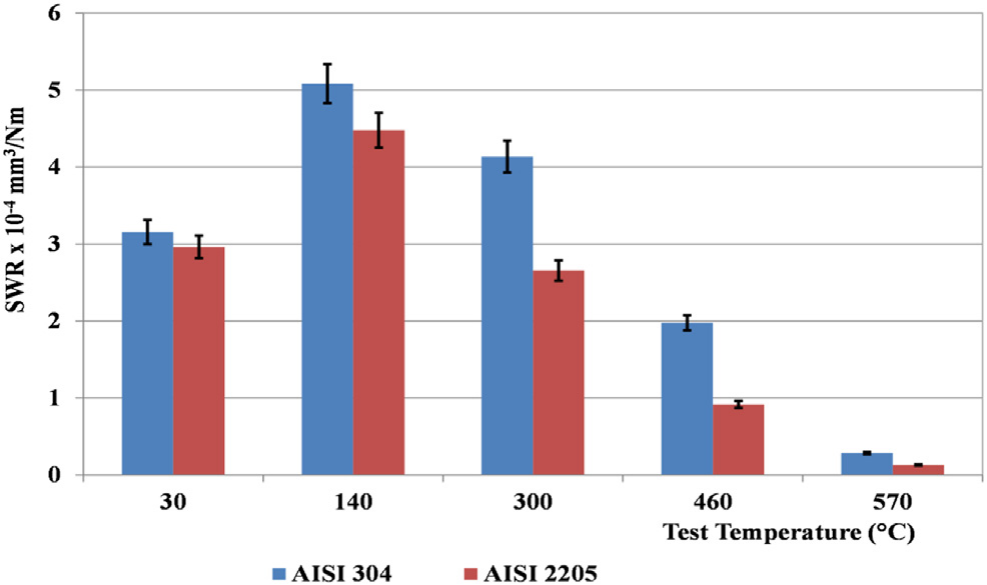
Effect of temperature on the specific wear rate of AISI 304 and AISI 2205 austenitic and duplex stainless steel at 45 N, 1.34 m/s, and 2000 m.

The specific wear rate which was calculated according to Eq. (1), initially increased rapidly with increasing temperature and the maximum occurred at 140 °C for both AISI 304 and AISI 2205. Thereafter, at 300 °C, the SWR of both alloys began to decrease continuously as the temperature increased, and the minimum wear rate for both alloys was at a temperature of 570°. The most obvious finding from the analysis is that AISI 2205 has a slightly higher wear resistance than AISI 304 at room temperature, while it is significantly higher at high temperatures. At room temperature, the SWR of AISI 2205 was only 6% lower than that of AISI 304. At 460 °C and 570 °C, this ratio exceeded 50%, furthermore, the SWR of both alloys decreased sharply. The maximum standard deviation of the wear test results was up to 7%, as shown in Figure 2. In addition, ANOVA analysis was used and found that the p-value for temperature in both alloys was zero, indicating the significance of the temperature. The maximum contact temperature (T_max_) of pin surfaces during wear tests of AISI 304 and AISI 2205 was highly close to each other, therefore they were averaged, as shown in Table 1. At room temperature, the difference between Tmax and ambient temperature was 188 °C and decreased significantly up to 101 °C at 570 °C. The decrease in temperature difference was due to decreases in friction and wear with increasing test temperature due to the formation of oxide layers.

### 3.2 Worn surface analysis

**Figure 3:**
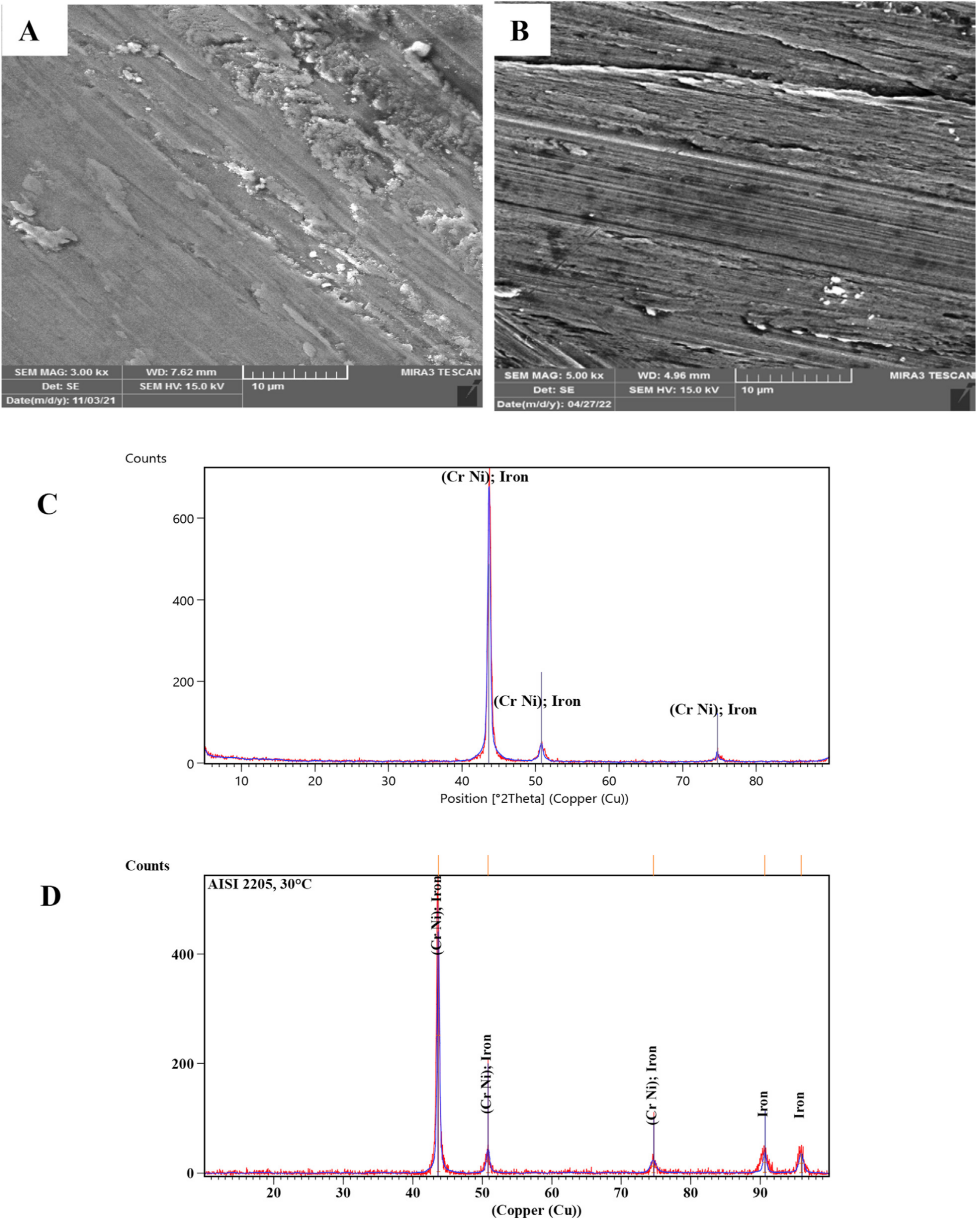
SEM image with a magnification of 3 KX for A) AISI 304 and B) AISI 2205 and XRD peaks of worn surface of C) AISI 304 and D) AISI 2205 at 1.34 m/s, 45 N, 2000 m, and 30 °C.

**Figure 4:**
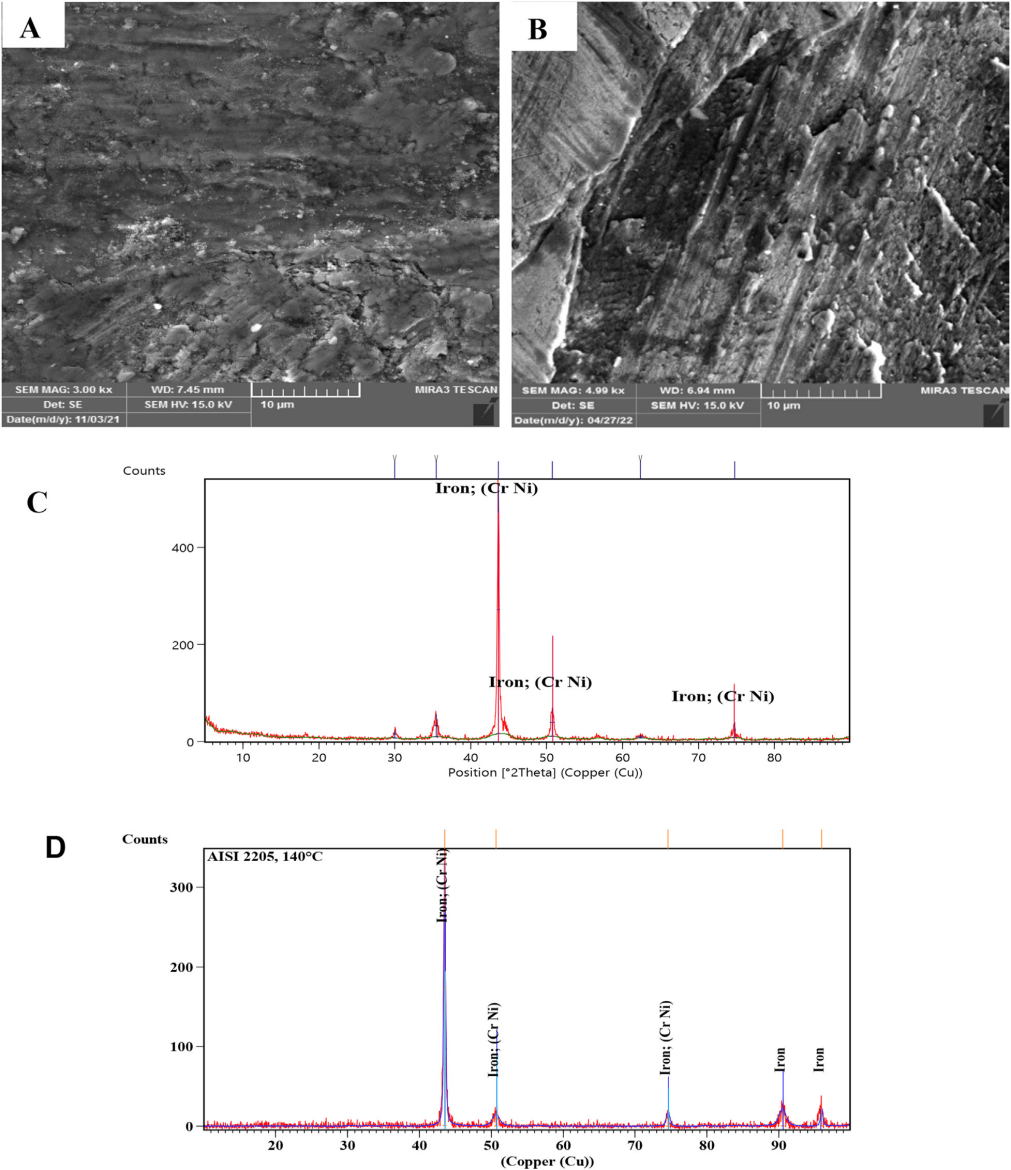
SEM image with 3 KX of A) AISI 304, and B) AISI 2205 and XRD peaks of worn surface of C) AISI 304 and D) AISI 2205 at 1.34 m/s, 45 N, 2000 m, and 140 °C.

**Figure 5:**
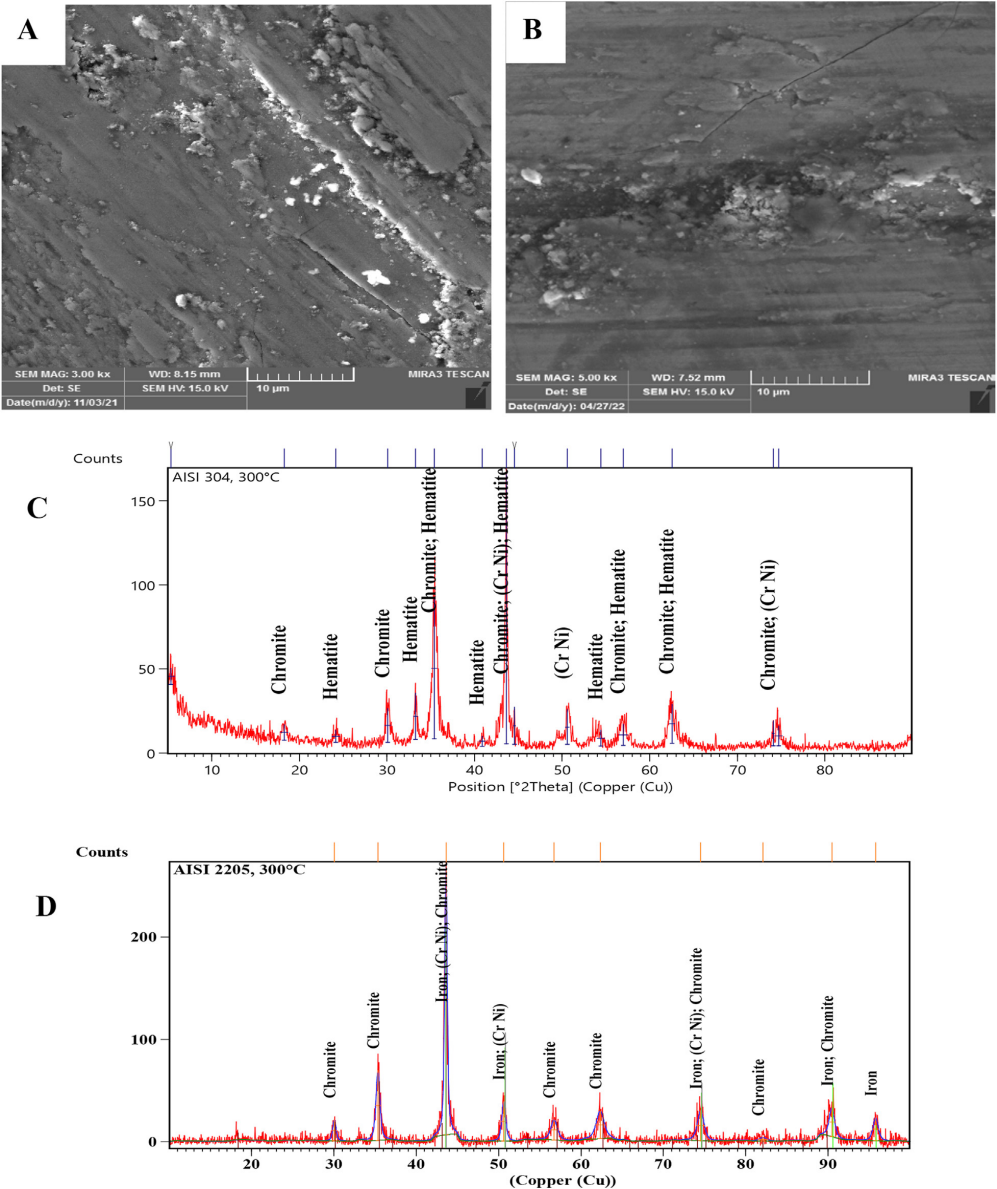
SEM image with 3 KX of A) AISI 304, and B) AISI 2205 and XRD peaks of worn surface of C) AISI 304 and D) AISI 2205 at 1.34 m/s, 45 N, 2000 m, and 300 °C.

**Figure 6:**
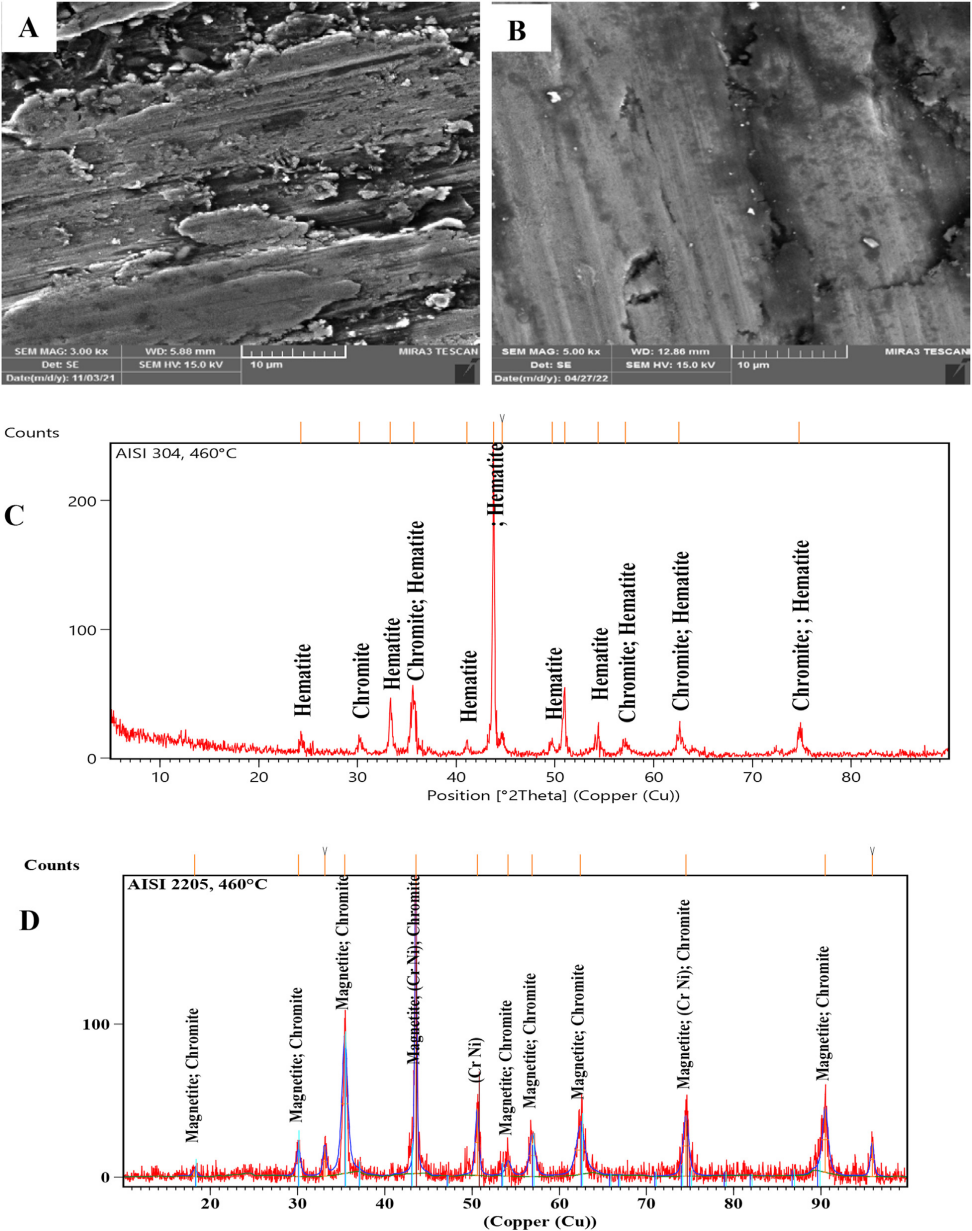
SEM image with 3 KX of A) AISI 304, and B) AISI 2205 and XRD peaks of worn surface of C) AISI 304 and D) AISI 2205 at 1.34 m/s, 45 N, 2000 m, and 460 °C.

**Figure 7:**
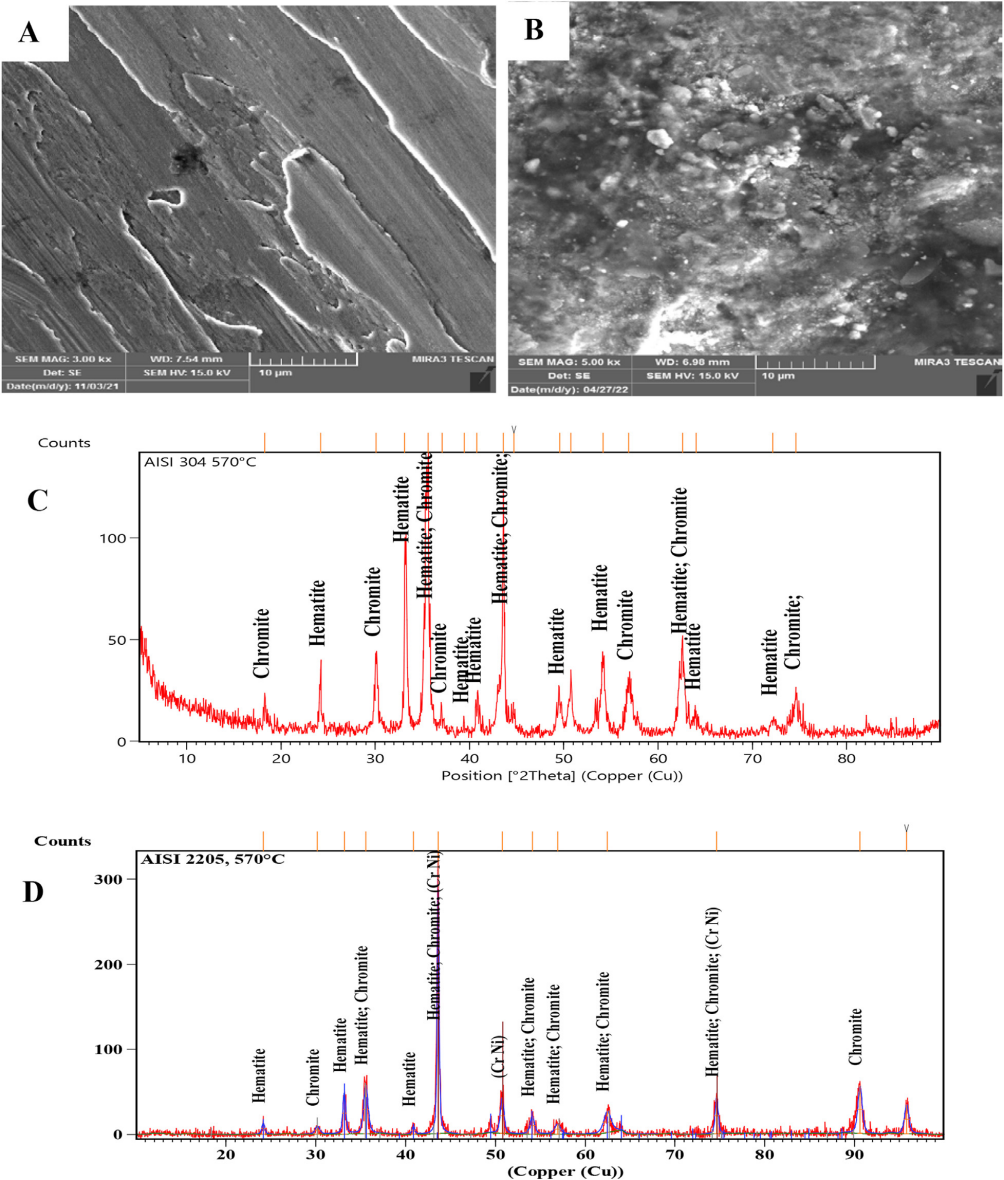
SEM image with 3 KX of A) AISI 304, and B) AISI 2205 and XRD peaks of worn surface of C) AISI 304 and D) AISI 2205 at 1.34 m/s, 45 N, 2000 m, and 570 °C.

SEM Images with a magnification of 3.00 KX and the XRD technique of the worn surfaces of AISI 304 and AISI 2205 were used to analyze the morphology and composition of the worn surfaces tested at different temperatures. At room temperature, the surface of AISI 304 was scratched and direct grooves peeling and plastic deformation occurred. However, only direct deep grooves were observed on the worn surface of AISI 2205, as shown in Figure 3-A and B. Figure 3-C and D present that the XRD peaks of both alloys are similar and there are no oxide phases. Sever worn surfaces with deep grooves, plowing and plastic deformation occurred on both surfaces in Figure 4-A and B that tested at 140 °C, as well as cracks and holes, appeared on the surface of AISI 304. As in the tests at room temperature, the XRD peaks consist of the composition of the alloys without the formation of oxides, as can be seen from Figure 4-C and D. At 300 °C, oxides began to form on the worn surfaces of both specimens. Fewer grooves and plastic deformations were observed on the surface of AISI 304 than in the previous tests at lower temperatures, but lighter grooves and fewer plastic deformations occurred on the surface of AISI 2205, as shown in Figure 5-A and B. XRD analysis showed the formation of chromite and hematite in AISI 304, while chromite was formed on the surface of AISI 2205, as shown in Figure 5-C and D. In addition to the formation of oxide layers, as shown by the XRD analysis of both alloys in Figure 6-C and D, deep grooves, cracks, holes, and plastic deformation appeared on the surface of AISI 304 that tested at 460 °C, while lighter grooves appeared on the oxidizing surfaces of AISI 2205, as shown in Figure 6-A and B. At 570 °C, the surfaces of AISI 304 and AISI 2205 were completely covered with oxides such as chromite, hematite, and iron oxides, as confirmed by the XRD results, (see Figure 7-C and D). Furthermore, plastic deformation, exfoliating, cracks, and holes appeared on the surface of AISI 304, while cracks and minor plastic deformation were observed on the surface of AISI 2205 (see Figure 7-A and B).

### 3.3 Worn surface microstructure

**Figure 8:**
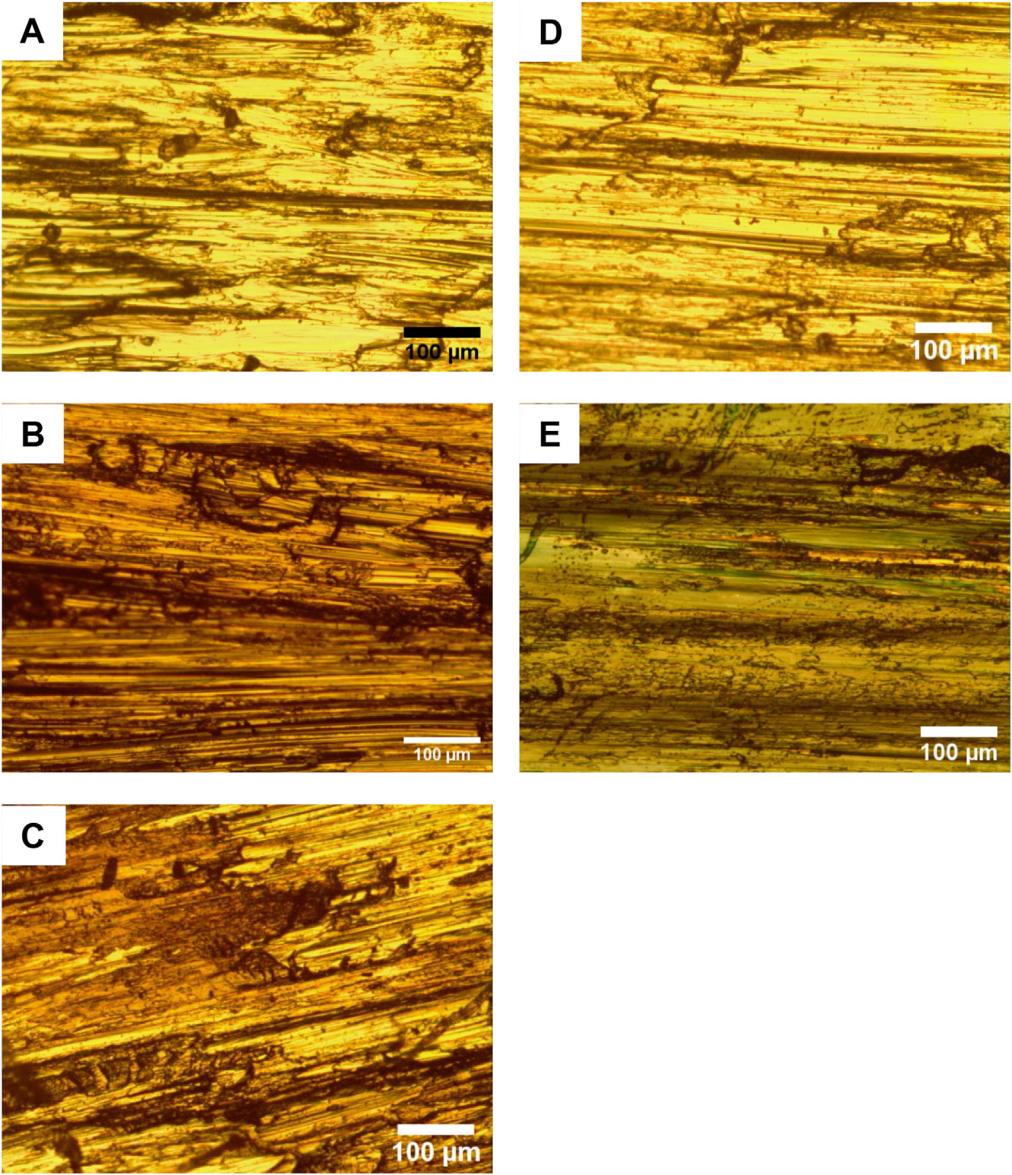
Microstructure of worn surfaces of AISI 304 wear tested at 45 N, 1.34 m/s, A) 30 °C, B) 140 °C, C) 300 °C, D) 460 °C, and E) 570 °C.

**Figure 9:**
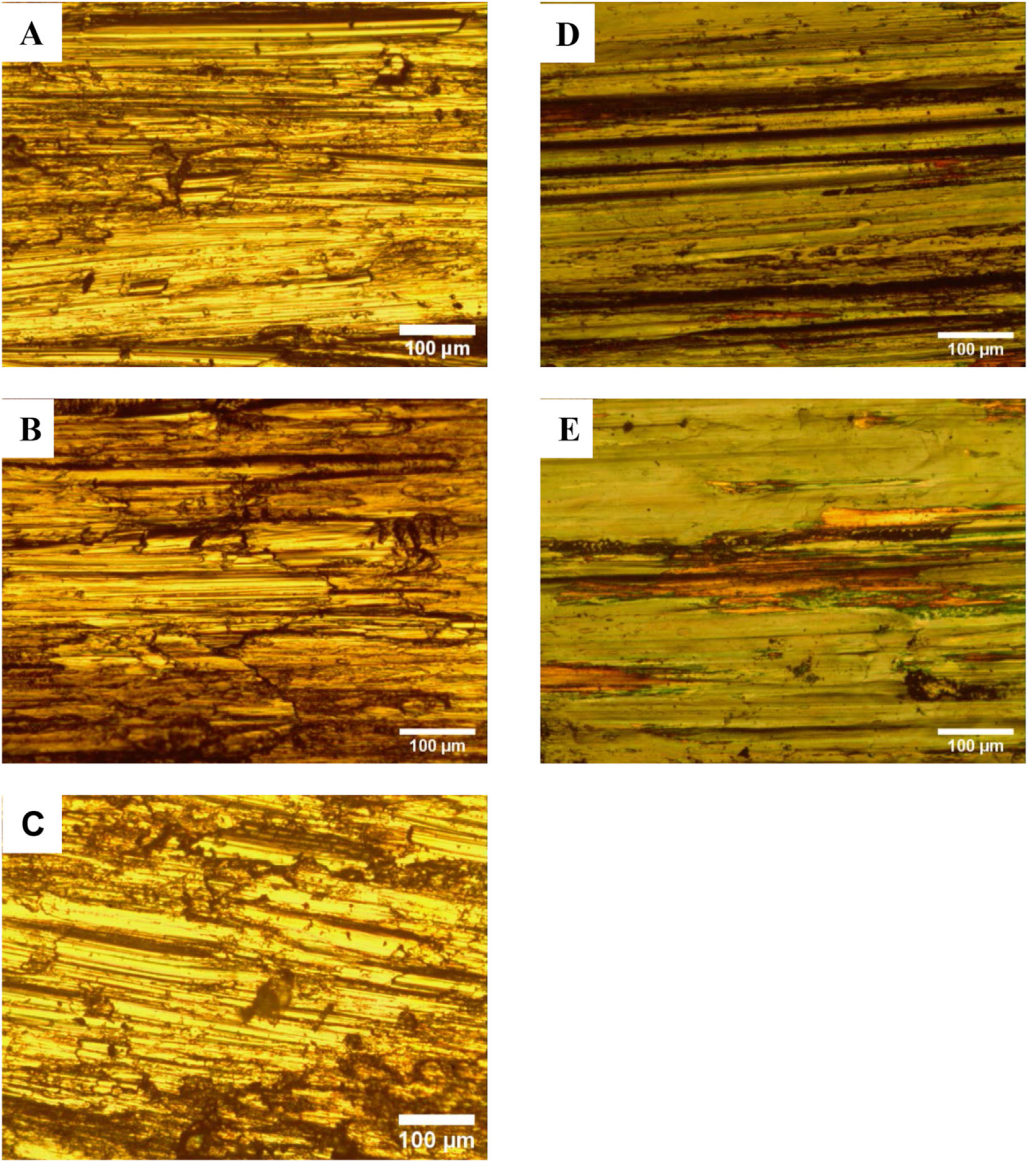
Microstructure of worn surfaces of AISI 2205 wear tested at 45 N, 1.34 m/s, A) 30 °C, B) 140 °C, C) 300 °C, D) 460 °C, and E) 570 °C.

Optical microstructure at 100x magnification was used to show the microstructure of the worn surfaces after testing. Figures 8 and 9 show the microstructure of AISI 304 and AISI 2205 specimens tested for wear at various temperatures. The high similarity of images of surfaces tested at the same temperature for both alloys. The traces are mostly abrasion wears such as grooves and plastic deformation. The most distorted surfaces are those of pins tested at 140 °C, while samples tested at 300 °C and above show a change in surface color due to the formation of oxides. The surface of pins that tested at 570 °C are less deformed and wear is minimum here.

## 4 Discussion

Table 2 and Figure 1 show that AISI 2205 has higher Cr and Mo content than AISI 304, which contains more Ni, and that the ferrite content in AISI 2205 is 52% while in AISI 304 it is only 18.7%. Therefore, the ultimate strength and hardness of AISI 2205 are significantly higher. These results are consistent with recent studies indicating that Cr and Mo promote the stabilization of ferrite, while Ni stabilizes austenite, and the ferrite phase has higher hardness and strength (Jebaraj and Ajaykumar, 2013; Rajkumar et al., 2021).

Figure 2 shows that the specific wear rate of AISI 304 and AISI 2205 was partially identical at room temperature. However, in other tests conducted at higher temperatures, there was a significant difference between them. The SWR of AISI 304 was 12% higher than the SWR of AISI 2205 at 140 °C, increased to 35% at 300 °C, and then doubled at 460 °C and 570 °C. The high wear resistance of AISI 2205 compared to AISI 304 is attributed to its high strength and hardness. This is due to the high ferrite content in the duplex alloy.

The specific wear rate increased by more than 50% for both alloys as the temperature increased from room temperature to 140 °C, since an increase in temperature leads to a decrease in hardness and strength, as confirmed by Hernandez et al. (2014), Hernandez et al. (2015) and Alvi et al. (2020). At 300 °C, the formation of oxides started, as shown by the XRD peaks, however, mechanical properties decreased more, but the wear rate decreased compared to 140 °C. The decrease in SWR continued with increasing temperature up to 570 °C, due to the formation of more oxides on the worn surfaces. This is because oxide phases have high hardness and act as a self-lubricant that reduces friction (Wang et al., 2019; Huang et al., 2020).

The nearly identical contact surface temperature for both alloys suggests that the contact temperature and heat generation are influenced by the operating conditions rather than the stainless steel grades. The XRD technique showed that the formation of oxides started at 300 °C, which is, however, a low temperature for oxide formation, as shown by previous work, e.g., in Parthasarathi and Duraiselvam (2010) oxides started at 350 °C and in Huang et al. (2020) at 400 °C. In this work, when the test temperature was 300 °C, the contact surface temperature was 450 °C and the SEM images of both alloys showed that the surfaces were partially covered by oxides. Next, at 460 °C, the wear rate decreased significantly as larger areas of the surfaces were covered with oxide layers as the contact temperature exceeded 560 °C, and traces of wear and remaining deposits were observed. Finally, at 570 °C, the contact surface temperature was 671 °C, so the surfaces of AISI 304 and AISI 2205 were completely covered with oxides, making the surfaces hard and well self-lubricating, so the wear rates were minimal and partially equal. In all experiments, the surface of AISI 304 was more worn than that of AISI 2205, and the material removal rate and wear rate were also higher. Consequently, AISI 2205 is a reasonable and preferable substitute for AISI 304 in important applications and especially at high temperatures.

## 5 Conclusion

The present research aimed to investigate and compare the SWR of AISI 304 and AISI 2205 at different temperatures. This study has yielded the following observations and results:

1. The SWR of AISI 304 is slightly higher than that of AISI 2205 at room temperature, and the difference increases with increasing temperature. At 460 °C and 570 °C, the SWR of AISI 304 is more than twice that of AISI 2205.
2. SEM images show that the AISI 304 specimens are more worn than the AISI 2205 specimens under the same wear conditions, with deeper grooves, more plastic deformation, and exfoliating.
3. Initially, the SWR of both alloys increases with increasing temperature and then starts to decrease at 300 °C due to the formation of oxide phases in both alloys. As the worn surfaces are completely covered by oxide layers at 570 °C, the SWR of both alloys drops to less than 10% compared to that at room temperature.
4. SEM images and XRD analyses show that the oxide phases start to form at 300 °C, but in reality, the contact temperature was 450 °C.
5. The oxide layers act as a protective glaze layer on the worn surfaces of the stainless steel grades and help to significantly reduce the wear rate.
6. AISI 2205 is recommended for special applications in high temperatures up to 500 °C instead of the austenitic stainless steel AISI 304.

## Declarations

### Author contribution statement

Dler A. Ahmed: Conceived and designed the experiments; Performed the experiments; Wrote the paper.

Mohammedtaher M. Mulapeer: Analyzed and interpreted the data; Contributed reagents, materials, analysis tools or data.

### Funding statement

This research did not receive any specific grant from funding agencies in the public, commercial, or not-for-profit sectors.

### Data availability statement

No data was used for the research described in the article.

### Declaration of interest’s statement

The authors declare no conflict of interest.

### Additional information

No additional information is available for this paper.
